# Symptomatische Hodenmetastase eines azinären Adenokarzinoms der Prostata

**DOI:** 10.1007/s00120-020-01194-w

**Published:** 2020-04-04

**Authors:** L. Kollitsch, C. Hamann, S. Knüpfer, D. Meyer, P. Kneissl, E. Jüttner, D. Osmonov

**Affiliations:** 1 Klinik für Urologie und Kinderurologie, Universitätsklinikum Schleswig-Holstein-Campus Kiel, Kiel, Deutschland; 2 Institut für Pathologie, Universitätsklinikum Schleswig-Holstein-Campus Kiel, Kiel, Deutschland

**Keywords:** Prostatakarzinom, Metastasiertes Prostatakarzinom, Tumorprogression, Tumormigration, Seltene Metastasen, Prostate cancer, Metastatic prostate cancer, Tumor progression, Tumor migration, Rare Metastases

## Abstract

Wir berichten über den äußerst seltenen Fall einer symptomatischen testikulären Metastasierung durch ein azinäres Adenokarzinom der Prostata. Bislang wurden Hodenmetastasen von Prostatakarzinomen (PCa) meist nur zufällig im Rahmen von therapeutischen Orchiektomien oder Autopsien entdeckt. Insgesamt wurden in der Literatur nur wenige klinische und v. a. symptomatische Fälle beschrieben. Trotz des raren Auftretens von Hodenmetastasen sollte bei fortgeschrittenen PCa differentialdiagnostisch an die Möglichkeit einer solchen Metastasierung gedacht werden.

## Abstract (en)

We report about the rare occurrence of symptomatic testicular metastasis of an acinar adenocarcinoma of the prostate. Testicular metastases are usually incidentally detected in patients treated with bilateral orchiectomy or more often during autopsy. In the literature, there are only a few clinical cases describing symptomatic testicular metastases. However, the possibility of such metastases should be considered in patients diagnosed with advanced prostate cancer. Testicular examination should be performed regularly, even in patients with low prostate-specific antigen levels.

## Anamnese

Im September 2016 wurde bei unserem Patienten, 1941 geboren, ein azinär differenziertes Adenokarzinom der Prostata (T2N1M1) mit einem Gleason-Score von 5+4=9 bei einem initialen PSA-Wert von 43 ng/ml durch eine einseitige, ex domo durchgeführte Stanzbiopsie diagnostiziert. Die stattgefundenen Staging-Untersuchungen (CT und PSMA-PET/CT) ergaben multiple LK-Metastasen, beidseits iliakal bis 2,5 cm und beidseits paraortal bis 2,2 cm. Der Patient erhielt eine Androgendeprivationstherapie (ADT) mit Leuprorelinacetat ab Oktober 2016 (davor Bicalutamid) und schon im November 2016 kam es zu einem PSA-Abfall auf nur 0,81 ng/ml. Die MRT-Kontrollbildgebung bestätigte eine deutliche Größenreduktion der LK-Metastasen als auch der Prostata. Aufgrund des guten Ansprechens und nach Rücksprache mit dem Patienten wurde die ADT als therapeutische Hauptmaßnahme empfohlen. Der PSA-Wert wurde regelmäßig kontrolliert und hielt sich im Verlauf immer auf <0,1 ng/ml. Nachfolgende MRT- und CT-Kontrollen konnten keine pathologisch vergrößerten Lymphknoten, Knochenmetastasen oder Absiedlungen in anderen Organen detektieren. Der Patient entschied sich jedoch Anfang 2019 schlussendlich gegen das Abwarten auf eine eventuelle Progression des PSA-Werts und für die Durchführung einer definitiven perkutanen Radiotherapie seiner PSMA-positiven Manifestationen, die im Rahmen der Staging-Untersuchung 2016 entdeckt wurden. Nach einer CT-gestützten dreidimensionalen Volumenplanung erhielt der Patient (im Zeitraum von ca. 1 Monat) eine lokoregionäre Radiotherapie der primären Tumorregion sowie der pelvinen Lymphabflusswege mit einer initialen Dosis von 2 Gy und 15 MeV-Photonen bis zu einer Kumulativdosis von 74 Gy. Die paraortalen-LK wurden kleinvolumig mit einer Erstdosis von 2 Gy bis zu einer Enddosis von 64 Gy aufgesättig. Bei der Kontrolluntersuchung im April 2019 gab der Patient an, dass er die Bestrahlung gut vertragen habe und dass er bis auf Nykturie und kurzeitig auftretende Diarrhö keine weiteren Beschwerden hatte. Der Patient befand sich zu diesem Zeitpunkt in einem guten AZ (Karnofsky-Index von 90 %) und in normalem EZ. Die Haut befand sich im Bereich der Bestrahlungsfelder in einem reizlosen Zustand. Außer dem bekannten Vorhofflimmern, weswegen der Patient auf Xarelto eingestellt war, wies er keine weiteren Komorbiditäten auf.

## Befund

Im September 2019 stellte sich unser Patient mit seit 6 Wochen bestehender Verhärtung, Schwellung und neu aufgetretenen Schmerzen seines linken Hodens vor. In der Sonographie ließ sich eine 2 × 2 cm große echoarme, inhomogene, hypervaskularisierte Raumforderung im Unterpol seines linken Hodens darstellen. AFP, HCG, β‑HCG und LDH befanden sich im Normbereich. Sein letzter PSA-Wert betrug im August 2019 0,1 ng/ml.

## Diagnose

**Figure 1:**
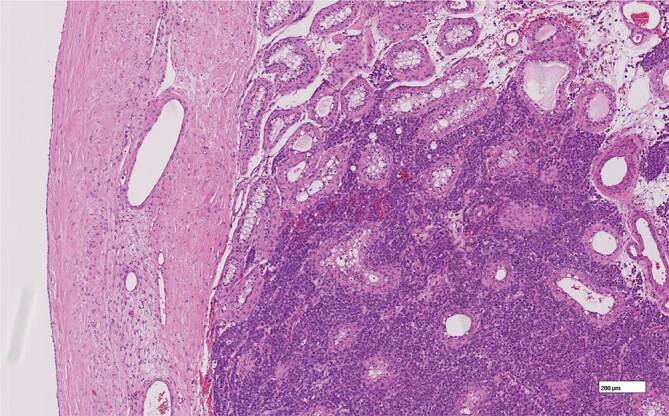

Durch eine erfolgte Ablatio testis links konnte eine 2,3 cm durchmessene Hodenmetastase eines azinär differenzierten Prostatakarzinoms (PCa) mit Infiltration des Rete testis histologisch, wie in Abb. 1, 2 und 3 erkenntlich, nachgewiesen werden.

**Figure 2:**
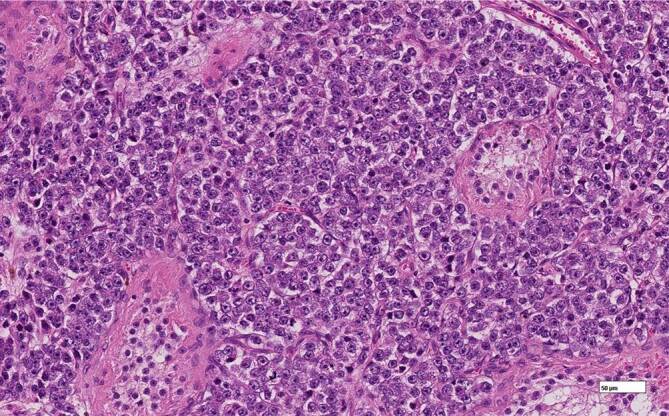

**Figure 3:**
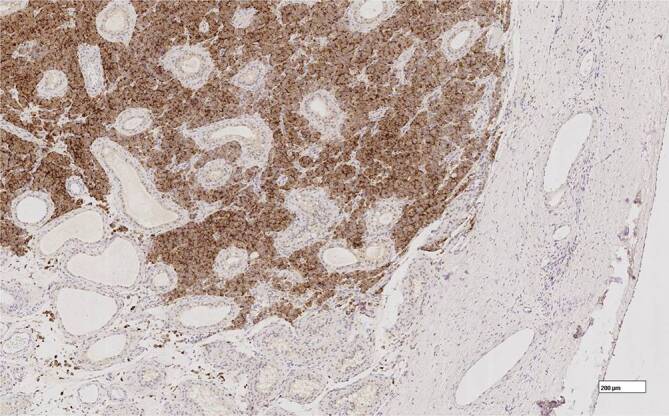

## Diskussion

Sekundäre testikuläre Neoplasien sind mit einer Inzidenz von 0,06 % insgesamt sehr selten, insbesondere mit in der Prostata lokalisiertem Primärtumor [6]. PCa präsentieren sich typischerweise mit Absiedlungen in den Knochen, Lymphknoten, der Lunge, Leber und/oder Gehirn [1]. Eine im Jahre 2000 veröffentlichte Autopsiestudie mit 1589 Patienten ergab, dass nur 0,5 % der Metastasen eines PCa in den Hoden vorkommen [1]. Johansson und Lannes fanden 1983 bei 4 % ihrer therapeutischen Orchiektomiereihe von 80 Patienten Hodenmetastasen [3]. Kirkali et al. berichten 1990, dass von 916 Patienten mit diagnostiziertem und therapierten PCa 124 (13,5 %) eine bilaterale Orchiektomie erhielten. 3 dieser Patienten (2,4 %) hatten Hodenmetastasen, einer davon bilateral [4]. Hodenmetastasen eines PCa sind meist asymptomatisch und werden überwiegend zufällig durch eine Autopsie oder nach bilateraler Orchiektomie zur hormonellen Tumorsuppression bei fortgeschrittenem PCa festgestellt [10]. Es sind nur wenige klinische Fälle dokumentiert, in Deutschland wurden bisweilen nur 4 Fälle publiziert [2, 7–9]. In der Literatur wird über verschiedene mögliche Metastasierungswege diskutiert: retrograd venös, retrograd lymphatisch, arteriell embolisch, retrograd über den Ductus deferens oder durch direkte Ausbreitung [7]. Tu et al. formulierten die Hypothese, dass gewisse Subtypen von duktalen und endometrioiden Adenokarzinomen der Prostata aggressiverer Natur sein sollen und bevorzugt in die Hoden metastasieren [10]. In unserem Fall entstammte die Metastase von einem azinär differenzierten Adenokarzinom und äußerte sich in Form von Schwellung, Verhärtung und Schmerzen des Hodens. Da unser Patient mit einer ADT behandelt wurde kam es vermutlich zu keinem erwähnenswerten Anstieg seines PSA-Werts. Das Auftreten von Hodenmetasen bei PCa scheint aber ein schlechter Prognosefaktor und ein Zeichen einer bereits fortgeschrittenen Erkrankung zu sein [5].

## Fazit

Obwohl Hodenmetastasen bei PCa eine Rarität darstellen, sollte die Möglichkeit einer sekundären testikulären Metastasierung nicht in Vergessenheit geraten. Vor allem da therapeutische Orchiektomien aufgrund der Verfügbarkeit von LH-RH-Analoga kaum mehr durchgeführt werden. Auch bei niedrigen PSA-Werten sollten die Hoden regelmäßig kontrolliert werden.
